# “Enclave in Transition”: Ways of Coping of Academics from Ultra-Orthodox (Haredim) Minority Group with Challenges of Integration into the Workforce

**DOI:** 10.3390/ijerph17072373

**Published:** 2020-03-31

**Authors:** Tehila Kalagy

**Affiliations:** Department of Public Policy and Administration, Ben-Gurion University of the Negev, P.O.B. 653 Beer-Sheva, Israel; kalagy@bgu.ac.il

**Keywords:** ultra-Orthodox, minorities, workforce, stress, coping, conservatism

## Abstract

Traditional societies around the world face various challenges with the introduction of “modern” values as a result of various globalization processes occurring worldwide. In the research literature, these groups are generally referred to as a “transitional societies.” The focus of the research discourse on “a society in transition” is the social change derived from the undermining of that traditional society and the weakening of its constituent values with the acquisition of higher education and modification of traditional division of roles in the family. In the last two decades, the ultra-Orthodox society in Israel has undergone far-reaching changes that are reflected in the acquisition of higher education and the accelerated entry into the employment market. In light of these changes, this study seeks to examine how the academic ultra-Orthodox deal with this integration into a work place outside the “enclave.” Methodologically, the study is based on qualitative content analysis of four focus groups, two for men and two for women, as is customary in ultra-Orthodox society. During the group discussion, participants were asked to describe how they cope with conflicts and their general professional challenges in the workplace. The findings of the study show that both the men and the women, described themselves as adaptable and coped well, despite the social difficulties facing their community and professional challenges in the employment space. The analysis of the major themes relies on the Stress and Coping theories.

## 1. Introduction

A conservative community frequently faces two major dilemmas: should it open to the external environment and its effects, and if so, to what extent? Both dilemmas stem from the central ambition of the divergent enclave to preserve its conservative values [1,2]. However, community members, whose existence depends on external society, are debating whether and how they can combine the “outside” and the “inside” without compromising the values of the community. In this context, they are very much concerned with their proper attitude toward acquiring an academic education, which is the salient manifestation of this combination, since acquiring higher education, and then a job, could potentially create a value conflict between the conservative Torah world and the changing modern world [3], thereby undermining stability. During the 20th century, globalization processes were expanded, as well as markets offering a variety of modern cultures and styles. This global media trend has, to some extent, forced conservative societies to open up and encompass a variety of concepts [1,4]. Conversely, traditional conservative societies often feel hostile to trends that offer diversity, change, and materialism that undermine spiritual life and tradition. The same conservative groups have had to deal with mass media, accelerated computing and the internet, penetrating and undermining the boundaries of the “enclave” and the existing traditional social order. Secular modernity combined with state-of-the-art technology, with its lack of values, posits man as an individual, free from any tribal and traditional affinity [3,5,6,7].

This threatens the leadership’s position and triggers a negative reaction to those who have chosen a different way in the community. Negative attitudes toward non-traditional education and employment processes place the individual in ongoing conflict situations and make it difficult to integrate. Moreover, it may affect the legitimacy of the individual in the community to which it belongs.

The current study sought to examine the ways in which the academic ultra-Orthodox deal with the “outside” during the integration into the employment market. The phenomenological analysis of the research materials was done in light of the interaction model for coping with the pressure by Richard Lazarus and Susan Folkman [8]. The two researchers have tried to answer how people deal with stress situations given their personal characteristics and, of course, the characteristics of the particular situation in the context in question.

## 2. Conceptual Framework and Theoretical Background

### 2.1. Transitional Society

A key term used in the research literature in the context of describing traditional societies is “transitional society.” This term combines two seemingly contradictory theoretical terms—traditional society and modern society. Traditional society is characterized by strong emotional ties between its members and a strong sense of belonging to the community and family. Formal education is not so important to it and is characterized by conservative religious traits, which are reflected in traditional employment patterns [9]. The focus of the research discourse on “transition society” is the social change derived from the undermining of the traditional structure of the ultra-Orthodox society and the weakening of constituent values such as patriarchal family structure and traditional division of roles in the family.

Societies undergo processes that affect their identity, following the infiltration of new values, such as acquiring higher education and then integration into an out-of-community workplace. In Israel, conservative minority groups are generally considered “transitional societies,” as is the ultra-Orthodox society. Proponents of modernity assume that modernization of the individual and society is made possible by processes, one of which is an increase in the level of education and changing employment patterns of men and women. These processes inevitably affect the individual, the family, and society, when combining dominant cultural values, with those of their own culture [10,11].

### 2.2. Ultra-Orthodox Society—An Enclave in Transition

An “enclave in transition” is a phrase that came up in the current study, which expresses in broad terms the changes that are taking place in Israeli ultra-Orthodox society in its current form [12]. The combination combines two common terms in the study of traditional minority groups. The first term is “transitional society” as described above, and the second, “enclave culture,” rests on Douglas’s theory and expresses the concept of the seclusion of conservative societies [1,2]. In this context, most researchers ask about the identity of the enclave and their ways of dealing with the impact of global changes on conservative societies in general and on ultra-Orthodox society in particular. One of the significant changes in modern society is the need to acquire education for occupational integration.

Collins (1979) [13] defines the social necessity of acquiring “qualifications” in order to integrate into the modern society that is defined as giving the qualifications. In ultra-Orthodox society, acquiring an academic education is an act that, from a value point of view, has not yet received general public consent and is, from a practical point of view, full of difficulties. One of the key issues that the ultra-Orthodox society is debating is the proper attitude toward acquiring a general education—which is one of the characteristics of the “external” society—because it also serves as a source for creating and disseminating modern values and is at odds with the value of community self-preservation [14]. Although the acquisition of higher education by the ultra-Orthodox, and especially by women, enjoys some legitimacy in the community, many of the rabbis and a large part of the ultra-Orthodox public still boycott it.

As mentioned previously, the ultra-Orthodox community in Israel has undergone a significant process in the past two decades in relation to the world of employment and the acquisition of higher education—there has been a significant increase in the number of ultra-Orthodox students studying in academic and professional institutions, as opposed to the past [15]. The escalation in the numbers of ultra-Orthodox learners in academic institutions, and working is due to a number of factors: the penetration of modern norms into the ultra-Orthodox community, worsening economic distress in this community due to cuts in allowances and the significant reduction in contributions from abroad, the culture of abundance, and an increase in living standards. Another factor that is of particular concern to ultra-Orthodox women is the change in the status of the teaching profession, which was until recently the main source of livelihood for them [16].

Integrators face complex barriers during entering the employment market. In the work space itself, they have to deal with issues related to being religious people, such as men and women working together, prayer times, and kosher food. In addition to all of these, there are cultural gaps that make it difficult to socialize with other employees as well as lack of professional knowledge. The men, in particular, have difficulty interacting with technology (computer use, etc.) and often lack English language knowledge [16].

In addition, they have to deal with conflicts in the family–community space—for example, the need to confront the ideal of segregation and seclusion from Western culture and to continue working in adult life within the community according to customary norms.

The purpose of the present essay is to examine how academic ultra-Orthodox deal with stressful situations in an employment space outside the enclave. This may help to formulate ways for the best integration of secluded minorities into the general employment space, and the integrators may be a model for the rest of the community, a model that symbolizes coping capacity in a different environment from their community.

### 2.3. Coping with Stress and Conflict Situations

The dialectical process of the departure of ultra-Orthodox from their known boundaries into the “outside” world of employment is accompanied by practical and emotional conflicts. The hopping between a religious and closed conservative society and a non-religious liberal society poses complex challenges for the ultra-Orthodox and can often present conflict in their workplace. Socially, his employers and co-workers expose him to relationships and emotional and social interactions he did not know before. This new environment encourages him to assimilate the importance of emotional flexibility and adapt to social and personal stressors [12].

Coping is the realization of behaviors that are the product of assessments the individual makes about situations he experiences. These behaviors do not appear in a vacuum but are the result of a process that began even before encountering the circumstances. For example, recognizing that a response (as opposed to a lack of response) is available to the individual and has the power to recreate the feeling as less threatening than it appears at first glance. In another example, if a response (versus a lack of response) does not help to the extent that the individual expected it to help, it is possible for him/her to reassess the level of threat or the effectiveness of his response.

The complex system known as the “coping process” is a continuous cycle of the individual’s interaction with stress situations. As we mentioned above, there are two main coping strategies. The first is problem-focused coping—taking action to solve the problem. That is, the individual needs to do something to reduce the stress. The other is emotion-focused coping—managing the emotions that arise in response to the situation [8]. Although stress situations usually produce both types of strategies, the problem-focused strategies are usually the more dominant ones in that one feels able to do something meaningful and constructive in order to effect change. In contrast, the emotion-focused strategies are more dominant when one feels that the stress situation is unchangeable and hence provides no choice other than enduring it.

The interactional model for studying stress situations by Lazarus and Folkman (1984) [8] addresses the question of how individual and specific characteristics affect coping situations. Stress status, according to this model, is a subjective phenomenon. Each person’s response depends on how much he or she considers the situation as threatening or challenging and the coping resources available to them in dealing with the experience. Lazarus and Folkman’s approach [8] relies on the assumption that coping with stress is an active process associated with cognitive, emotional and behavioral aspects. External coping resources, such as social and family support, are of great importance for successfully coping with stressful situations. That is, positive relationships make a big contribution to developing coping strategies, expanding employee emotional resources, and effectively coping with stress and conflict situations.

Lazarus and Folkman distinguish between two types of coping strategies—problem-solving and emotion-focused coping. The problem-solving strategies obviously focus on solving the problem, while the emotion-focused strategies are centered on one’s attempt to change one’s own perception. Problem-solving strategies include, for example, planning or addressing religion or social support. Emotion-focused strategies involve, for example, venting emotions, self-blame, or denial [17].

The current study sought to examine the ways in which the academic ultra-Orthodox deal with the “outside” during the integration into the employment market. To address this, three key questions were defined:

(1) What strategies do ultra-Orthodox academics use in the realm of employment outside their community?
(2) Is there a difference in the coping patterns and coping resources of the ultra-Orthodox who remain in the ultra-Orthodox enclave compared to those who work “outside”?
(3) Are there differences in coping patterns between women and men?

The phenomenological analysis of the research materials was done in light of the interaction model for coping with the pressure by Richard Lazarus and Susan Folkman [8]. The two researchers have tried to answer how people deal with stress situations given their personal characteristics and, of course, the characteristics of the particular situation in the context in question.

## 3. Methodology

### 3.1. Research Procedure

The research was conducted for the Israel Democracy Institute and received the approval of the Ethics Committee for the Dispute Management and Conflict Program at Ben Gurion University. The participants were informed that we are interested in understanding their perceptions and opinions on the subject under study. The confidentiality of the work was also emphasized.

Participants were selected using the snowball sample method [18]. The decision to use this technique arose from the difficulty of accessing likely subjects, in particular those belonging to closed religious communities that prefer to avoid exposure. Such subjects are objectively difficult to reach due to their insulation [19]. The choice of this sampling method was also influenced by the suspiciousness characterizing religious communities and “hidden populations” that are difficult to locate and engage [20].

The research leans on the qualitative paradigm that claims to understand the phenomenon being explored in its daily natural environment [21], thus gaining insights into their experiences and meaning [22].

The data analysis was based on the “Grounded Theory” approach [23], which is relevant to research and raises general questions, as in the present study. This approach assumes that people with shared life circumstances also have common social and psychological patterns that grow out of their shared experiences. Accordingly, the researcher attempts to identify these key patterns and describe them in order to explain the phenomenon under study. The analysis according to this approach includes two steps: first, general topic analysis, which looks for key themes and patterns that emerged in the interviews; and second, providing an interpretation of those themes and the hidden and implicit meanings of the more visible ones.

### 3.2. The Focus Groups

In order to examine the central issue regarding the ways of dealing with the ultra-Orthodox, in the realm of employment, the central tools used in this study are focus groups. These groups were in fact a form of focus groups, whose central purpose was to explore the participants’ personal opinions and understand their feelings in depth [24].

The focus groups have a clear advantage of the dynamics between the participants which can reveal fascinating information about them. This is information that individual interviews will not provide. The same dynamics and sharing produce a diverse discourse, different from any other research tool. In addition, within a group, a focus on listening and special diagnostic ability is required. It requires the researcher to develop listening and analysis skills. In women’s groups, the group seems to be productive and unique in that it helps to address significant social issues that are sometimes taboo or met with silence [25].

Four focus groups were convened throughout December 2015. The groups were divided by gender, as is accepted in ultra-Orthodox society; 15 women (Table 1) and 11 men between the ages of 20 and 56 participated in the groups. The participants had varied occupational backgrounds (e.g., municipal management, teaching, software engineering, swimming instruction) and also belonged to different streams in Orthodox society (e.g., Lithuanian, Hassidic, Mizrahi, modern). Furthermore, participants in the focus groups reported that they work in a variety of workplaces (16 employees at ultra-Orthodox workplaces, two employees at national religious workplaces, and eight at secular workplaces).

The focus groups were recorded with the permission of the participants and then transcribed into text. In the article, all original names have been replaced with pseudonyms. The accumulated findings were interpreted through content analysis of the statements made by the subjects and used to construct the central themes presented below. The group discussions focused on the issue of coping with challenges of integration into the workforce.

## 4. Findings

The research materials relate to three main questions that the research sought to examine—What coping strategies do ultra-Orthodox academics use in the employment space outside their community? Is there a difference in the patterns and coping resources of the ultra-Orthodox who remain in the ultra-Orthodox enclave compared to the ultra-Orthodox who move outside? Are there differences in women’s and men’s coping patterns?

In general, the research materials indicate two major trends: first, the formulating of unique coping patterns of most participants with conflicts that have arisen in their work situation. The second, the use of problem-focused strategies as opposed to the use of ineffective (emotion-focused) strategies. I will address the three key questions posed by the research in detail.

### 4.1. Coping Strategies in the “Out-of-Community” Employment Space

The first research question, therefore, focused on examining the coping strategies that academic Ultra-Orthodox m take in their encounter with the “outside” working world, one characterized by modern secular values and culture. The different strategies have been grouped into two main strategies—adaptive (i.e., problem-focused) and non-adaptive (i.e., emotion-focused). The work place itself forces them to deal with conflicts associated with belonging to a conservative community with norms different from those outside. There are also work skills that the ultra-Orthodox lack due to their lack of experience.

#### 4.1.1. Instrumental Social Support

The coping strategy that stood out among the research participants was the appeal for instrumental social support. Joining other ultra-Orthodox workers in the workplace is a convenient and effective strategy for dealing with the conflicts that arise.

Participants’ comments reveal a combined strategy of seeking emotional support and a united format for solving problems. This form of coping stands out in workplaces with a number of ultra-Orthodox employees, who have gained power by instituting “ultra-orthodox” norms in the workplace, which help the integration of the ultra-Orthodox without harming the output of other workers.

*We do not come to an orientation day. They tried to persuade us in the past and tried to figure out why not. It includes accommodation, and we decided as a team that we’re not even giving it a chance, and they really respect that. And even if some of them curse, they really refrain to do so when we’re around. […] For example, there is a toast and they want us to come, so we come and everything is kosher by our standards, but we are observers and do not interfere as long as it is during work hours. But once there are fun days, etc., we don’t participate at all.*

Ruth speaks in terms of “they” and “us” as she describes her coping in the workplace. There is no doubt that social support from like-minded women serves as a pillar to help her personally cope with the difficulties but also provides a tool for dealing with employers when she and her friends present different demands.

Because it is a closed society, an ultra-Orthodox person is usually not exposed to his peers from the general population. As the ultra-Orthodox press (mostly printed, and also online) operates a strict filter of news and events in general society, ultra-Orthodox people are rarely exposed to behaviors and situations that are not appropriate for their community. We therefore asked how they emotionally cope with exposure to complex social situations. This question mainly answered by women who work in the fields of care and education in non-non ultra-Orthodox frameworks, as Michal replied:

*All in all … there are different views, so I’m not too involved. And I come from a home where my mother works and in the past, studied at the Hebrew University, where it’s really heresy, right against religion, and she always told us, “There’s nothing to do. We are in a world with lots of people and sectors and need to know how to cope.” I don’t intend to come and fight, I just keep to myself, the main thing is that as for moral deterioration, everything is okay, and parties and stuff I don’t interfere, and in opinions, too, I’m not fighting. I just keep to myself.*

#### 4.1.2. Dealing with Identity Dissonance

Zvika expanded on the difficulty of dealing with the complexity of maneuvering between the different identities—the private world versus the workplace.

*It’s not that you have to do something in practice, it’s just learning to live with that complexity. You need broad shoulders to learn to live with the conflict in this part, on this issue, of friends and identification. You have to learn to deal with the conflict, and may be harder for some and then they will have to make a change, maybe with the values or something.*

Zvika’s way of coping reflects another coping strategy that stood out in the results of the study—positive framing, i.e., the reading of the situation in positive terms. A positive interpretation helps manage the feelings of stress and not necessarily deal with the stress itself.

Most focus group participants demonstrated a positive worldview and described their workplace as positive, both at the interpersonal level of employers and employees and at the level of conditions and wages. They expressed high satisfaction with these variables, and the positive framing of the work was very prominent in their strategy of coping with the “outside.”

#### 4.1.3. Ways to Deal with the “Home vs Outside” Conflict

One of the challenges of a person who integrates into a workplace outside of his community is being in a space with a different value scale. This is especially the case for the ultra-Orthodox, a cultural minority group in Israeli society, since encountering different values of behavioral norms can create difficulty and even contradiction between the two worlds. Participants in the focus groups were asked, among other things, how they cope with a work space that has a different value scale from their own, in view of the fact that they come from a closed society that has advocated religious segregation and conservatism. The findings of the study indicate that the planning strategy is widely used, the most prominent of the strategies used by ultra-Orthodox academics. One of the difficulties that accompanies the ultra-Orthodox is the conflict between home and work. In this context, questions such as, Do I, and to what extent, share information with household members about my work? Will my integration in a non- ultra-Orthodox workplace negatively impact my personal status in the community? etc. Rafi describes his way of dealing with these aspects:

*I made a total separation between the two worlds. At work, even though I fit in there, I never associated with the employees, I never invited them to any event of mine, even if it might have been possible in some way. I made a total separation. Definitely, if you’re already raising the matter of matchmaking for example, they ask what the father does [...] I’ve never given exact details “teaches computers, deals with computers” ... it is referred to as “dealing with”. And even when I’m at work and all, I don’t try to bridge the two worlds, nor bring my world into work. On the contrary, maybe it is right or maybe wrong to do it. I try just to be ... And that’s it. Not trying to connect the two worlds and even trying to separate the two worlds.*

The new situation in Rafi’s life puts him in a work–home conflict in terms of values. His remarks reflect on his dilemma of how to combine the conservative values of home, with new values of his non-ultra-Orthodox workplace. Rafi takes the planning strategy, which means creating an action strategy idea that will take precedence over the action that should solve the problem. Rafi sees the difficulty in bridging the two worlds in which he operates and therefore “makes a total separation” between them. This strategy of action helps him move between the two poles and deal with the cultural and practical gaps between home and work. Rafi thus describes a kind of separation between the “part of the Torah,” namely his personal and family values, and that of dealing with the obligations of the workplace. The coping strategy is present in all focus groups as a common and unique coping strategy that creates a separation between the “outside” and the “inside.” The distinction between the private world and the surrounding world is thus a way of coping employed by many of the research participants in their encounter with the “outside” values.

The other strategy that has been very prominent among the participants is the active coping strategy, the one that takes practical steps. This strategy removes or bypasses the pressure factor and instead improves its results. Such direct actions characterize the focused approach to solving the problem. In each conflict raised for discussion, participants said they strive to work around the barrier or improve the situation.

### 4.2. Coping Methods of Employees outside the Enclave vs. Workers Inside the Enclave

#### 4.2.1. Employment outside the Community

The research materials show that there are real differences between those employed within the ultra-Orthodox community and those employed outside. The essential difference between the two groups of workers is the environment they need to deal with. While intra-community workers are not required to deal with heterogeneous and different societies, those who have been involved in work outside the community often face conflicts—both value conflicts and practical conflicts.

The voice of the participants in the focus groups reveals the professional challenge that exists in any workplace, but the struggle of coping with the value conflict in non-ultra-Orthodox workplaces is not felt at all by those employed in intra-community workplaces. The findings show that employees who choose to work in an ultra-Orthodox workplace do so deliberately because the key criteria for choosing this workplace rather than another are more important to them than the terms of wages, as Rachel demonstrates:

Over the years I have been looking for work. I see that it’s hard for me to be in a place that’s not ultra-Orthodox. It might be a kind of conflict. Even when I am offered a double salary or things like that, once it’s in a mixed environment, I stay where I am, I forget about it.

The thought of integrating in a place outside the community causes pressure among the integrators. There are those who have chosen to cope and integrate in a mixed place. However, there are those who plan ahead to integrate only in an intra-community work place. Planning brings up ideas for action and thinking about the steps to be taken to best address the problem. This activity inherently belongs to focused problem-solving strategies, but its distinctive feature is that it precedes the action of problem solving and actually belongs to the pre-action phase of the second assessment of the situation.

#### 4.2.2. Spiritual Coping

One of the main challenges the integrators face is in the spiritual aspect, meaning religious feelings and connection to the workplace. Those who integrate into community-based employment do not report significant spiritual difficulties, in contrast to those who have joined non-ultra-Orthodox places of employment. This inner conflict is expressed in a single word repeated in all focus groups—“toughening,” or as Shira expresses:

*I became tougher and more exposed. Obviously you don’t stay the same as in the seminar. I attended some group and one of my seminar teachers that was there had a student that “declined” spiritually while doing office work. She was righteous and yet “fell”, and then that teacher started a group on how to deal with office jobs, she prepared us and it helped. It’s a constant struggle. I make a strong separation […] there is a spiritual effect and that is a fact! Unless you come, do a quiet job and go without talking to anyone, it makes an impact and I’m not really a loner, but there is counterweight—on Shabbat I read books of morals and spiritual things that will strengthen me.*

The “toughness” is actually a coping strategy. Its use indicates that ultra-Orthodox people develop a defense mechanism, a kind of spiritual armor, when leaving for work outside. Taking this strategy helps them protect themselves from external influences.

#### 4.2.3. Traditional Clothing in a Secular Environment

Shira’s portrayal concerns spiritual aspects that can influence the level of religiosity of the integrators. Another aspect of religious norms is the traditional outer wear. The ultra-Orthodox population in Israel usually dress according to certain codes. Among men, this code is more pronounced. They wear a white shirt and a dark suit, and a brimmed hat. In this secular space this dress code is very unusual and prominent. It bothers the newcomers at first until they adapt to the place and are being observed less. There are also few who share that they come to work without their suit and hat, in order to stand out less.

Moreover, some feel that their usefulness at work is evident from Amos, who is a lawyer by profession. In his remarks, he expressed serious concern that his unique attire would endorse the stereotype that exists for the ultra-Orthodox, thereby damaging the client he represents:

*Before representation in law, there is always this fear of appearing as an ultra-Orthodox person. This … how did Oded tell me? If I show up with my beard, then I’m actually harming the customer. But when representing the client there is work needed to impress the judge and to gain his sympathy, despite the ultra-Orthodox appearance that I feel does not invite good treatment. Same thing just when I come with my suit and hat … I make sure to go with a hat and suit regularly.*

#### 4.2.4. Coexistence in the Employment Space

Most of the focus group participants reported that they felt that they were able to co-operate with common work norms on both sides. Compared to a closed ultra-Orthodox space that does not need coexistence, since the norms are monotonous and widely accepted. As Yaakov describes,

*The firm is mixed. It is secular, it also has quite a few ultra-Orthodox, but it’s mixed. And thank God, we manage […] to co-exist very well.*

The success of coexistence can be dependent on the fact that the integrators use adaptive strategies and seek practical solutions to all the conflicts that arise at work. From the second point of view, the participants say that employers address all the difficulties of the ultra-Orthodox workers and try to solve any conflict that arises in a practical way.

In sum, most participants take active coping and planning as coping strategies to deal with conflict situations in their workplace. Most of them stated that, on a personal level, they are paving the way for success and dealing with stress situations on a daily basis. Socially, most of them share the feeling that they are able to reach coexistence and mutual understanding with both their employers and their non-ultra-Orthodox colleagues in the workplace.

#### 4.2.5. Employment in a National-Religious Space

In spite of the dichotomy that we initially aimed for in the study, i.e., religious versus secular, another challenge was raised and that is working with people from the national-religious sector. As has emerged from all focus groups, the challenge of working with national-religious is the most difficult challenge, as it presents the most complex conflict situations to ultra-Orthodox workers, on the basis of the supposedly shared religious identity, as Shoshi describes,

*It was precisely there [in the secular workplace] that it was much easier for me to maintain my boundaries, because I think the differences were very clear, unlike today in the [accountants’] office where II work with a lot of religious people, and what is the difference between me and them?—“I’m religious too.” And sometimes I feel that it is wrong to present yourself as more religious than someone else because he is also strict about halacha, so you cannot tell him “it is wrong.” It is much easier to work there [in a secular workplace] than in a religious place, because there [in the secular workplace] my boundaries were stronger.*

The participants’ statements reveal the wide gap in Israel between the ultra-Orthodox and the national–religious society and teach that sociological differences are also important in the employment world. In fact, the participants’ statements indicate that conflicts on a religious cultural background arise not only between the ultra-Orthodox minority group and the secular majority group but also between the ultra-Orthodox minority group and another religious minority group. Indeed, ultra-Orthodox research participants also point out the difficulty of socializing with other workers who are national–religious in their workplace.

### 4.3. Comparing Women’s and Men’s Coping Strategies

The third question the study seeks to answer relates to the way in which men and women deal with employment. The main differences between men and women were found in their coping strategies—effective versus ineffective. Women use strategies of emotional support, instrumental social support, distraction, and emotion venting more than men do.

In the women’s focus groups, they talked more about the difficulties there and sought emotional support and a framework for expressing their feelings. In the men’s focus groups, the conversation focused more on the ideological level and the settlement of the contradictions between the world of employment and the ultra-Orthodox world. Hence, the ultra-Orthodox women feel less need than the ultra-Orthodox men to take into account the reactions of the environment to their departure for work, perhaps because they have been integrating into the general economy for years. The men, on the other hand, were already dealing with the environmental reaction regarding the ideological question of Torah study versus going to work. We note that in ultra-Orthodox society, men are commanded to study Torah and most totally aspire to it, compared to women who are required to support them. This “role reversal” may well explain the different ways of coping between women and men.

As mentioned, the family is an influencing factor on the integrators. Various studies have found that traditional families that embrace modern values have a major impact on the individual in coping with the adoption or rejection of those values, as well as in how she/he copes leaving the community for education or employment [12].

In the focus groups, men described the reality in which they must combine the ultra-Orthodox community and the family with the workplace, but their descriptions were characterized by striving to find practical solutions. Such as the solution of Moshe given below. Moshe describes the awkward conflict of exposing the children to the reality that Dad works in a non-religious place instead of studying Torah like the fathers of his children’s friends:

*Since I work where I work, I know there is life at home and at work and I don’t mix them up. When at home, I try not speak loudly on the phone so as not to change the atmosphere. Why? Because the kids are in a very specific framework and want to see that their dad is the same as their friends’ dad, and I don’t think that they should be exposed to this at their age. When they grow up they can do whatever they want.*

The women, on the other hand, used discussion in the focus groups as part of their strategy to get emotional social support in their trying coping efforts. At the end of the discussions, the women thanked us for the invitation to attend (despite the difficulty of attending them in the evening). They said the discussions game them food for thought about the process they were experiencing. They also offered to have such support groups, each in her own community, and reasoned that their identification with their peers’ difficulties helps them—both because they allow them to express their feelings aloud and because they help them find practical solutions to difficulties in the workplace.

A significant difference was found between ultra-Orthodox women working as a single individual in a foreign system and ultra-Orthodox women working together to form a cohesive group. In the public service, such organizing is particularly evident in the designated sections for ultra-Orthodox women, a kind of enclave. A similar arrangement also exists in the Jerusalem municipality, in one of the divisions, where it was not intended in advance to separate the ultra-Orthodox workers from the other workers, but in practice the ultra-Orthodox women were grouped together under a division manager who happens to be ultra-Orthodox herself, and also serves as their informal representative. The same division manager participated in one of our discussion groups, and the strategy she presented in her workplace is active coping, mostly receiving instrumental social support. She explained that her power to dictate norms in her department is based on the incorporation of the ultra-Orthodox employees, who together are empowered when facing the City Council.

Dina (division manager) said,

*These conflicts occur and I deal with them, my employees deal with them, I guide them how to deal with certain situations […] there are some kind of fun days with a program that I see are not appropriate for me. I inform the Division Manager that this is not right for me and we are not coming, and I back up my employees when they also decide not to come. I’ve created some kind of consensus when it comes to sports department, where there needs to be greater sensitivity. If there is a man in the environment who expresses himself in an improper or rude way, I am not embarrassed. It may not be for everyone. I get up and explain to him that we are married ultra-Orthodox women and we have children […] and do not talk in this way […] and they have learned to respect this over the years.*

#### 4.3.1. Lack of Professional Knowledge

One of the gaps in professional knowledge in the workplace is the knowledge of English. Moshe argues that the lack of knowledge of the English language eventually translates into a lack of professional knowledge, and in his words, “occupational disability.”

The lack of knowledge of the English language links the cultural gaps between the ultra-Orthodox society and the entire Israeli society, and this issue is more felt and spoken of by the men who are already in the work market or those who want to join it. This is undoubtedly one of the biggest barriers to entry into the labor force, especially among men, since they do not learn English in their study settings. Some have used a strategy of repression or reconciliation with the matter, and others have reported that they are trying to learn in parallel with working. The ultra-Orthodox men encounter the problem of lack of professional knowledge in their encounters with technology, computer use, etc. With this difficulty, they deal relatively easily, study in the workplace, and report a speedy closure of the gaps.

#### 4.3.2. Level of Religiosity

Women report a higher level of religiosity than men. Women also attach importance to the Halacha and hold views that are at odds with what is customary in their workplace. The feeling is that women are constantly trying to settle their conflicts with the “outside.” The men, on the other hand, feel relatively relaxed even though they are aware of the problems that arise when they work “outside” the community. There is also a difference between young women and older women in their religious-spiritual adaptation to the workplace. This difference is noticeable in the words of Rachel, the oldest participant in the group, who is already a grandmother:

*I was much more hysterical. I went through some complex situations. I am 25 years among these people. The people have changed, the dynamics changed and higher education has also allowed me to look at things differently. When I left the seminar I was a 19-year-old and came to the sports department and was scared of everything and came home pale and told my husband what I was going through. That’s no longer today because I have a bachelor’s degree in educational administration, we have experienced some insights, and we have acquired a master’s degree in conflict management and settlement, which in itself has given us insights into accepting the other without sacrificing our own principles. My sons and husband are “Talmidei Chachamim” (very learned), my kids study at holy yeshivas, and what shouldn’t come into our home doesn’t come in. But you really need some resilience. Do not be alarmed, live it without being hysterical and give it time.*

## 5. Discussion

The present research is of importance in the field of social policy and service planning for ultra-Orthodox society in particular and Israeli society in general. It is characterized by a particular observation of the researched community and the formulation of appropriate policy in accordance with the results of the qualitative research based on the voices of the participants in the various focus groups.

The empirical research materials combined with the theoretical aspects form the basis for the conceptualization strategies of members of the ultra-Orthodox community in their encounter with the “outside.” In other words, the participants’ comments allow for an overall view of ultra-Orthodox society in the context of changing processes in the employment space. It seems to us that a new model of working ultra-Orthodox people is emerging, one that adopts the integration strategy and at the same time practices different degrees of differentiation. In the working ultra-Orthodox community there is integration in the general society and there is a desire for them to be partners in the Israeli economy, and at the same time they draw an emotional and practical boundary line between themselves and the sector outside, and in this case, employers and their colleagues in the workplace.

Various studies that examine minority employment areas report primarily on a form of integration that does not recognize individual liberties and particular heritage. The present study is connected to other studies [26] that seek to assimilate the cultural complexity of integration and enable them to sustain their way of life in the workplace as well. Wasserman and Frankel argue that this is how we can ensure better integration of women along with maintaining their autonomy.

In fact, even when acquiring higher education, the enclave members are dismantling and reassembling modern values. In fact, the ultra-Orthodox integrators use problem-solving strategies as opposed to less effective ones. Using these strategies is a dual process of integrating into the majority group by adopting modern traits (higher education and employment) and strictly adhering to the traditional values of the minority group culture from which they come.

The ultra-Orthodox men and the ultra-Orthodox women adopt the integrated strategy differently. The academic ultra-Orthodox women, from the various streams, advocate conservatism and adherence to the values of the ultra-Orthodox community, i.e., Torah study for men and their children, and a desire for gender segregation. Women also rank their religiosity in a place higher than that of men, although they have been in the labor and education market longer than men and also define themselves as more open to different environments. Despite the acceptance of “outside” values by the women, they show stronger loyalty to the basic conservatism upon which they were educated. There are two distinct groups: integrating in mixed workplaces and maintaining their social religious values, and advocating segregated integration, i.e., claiming a homogeneous “enclave” in the work place that avoids social integration.

The use of adaptive strategies by most research participants is broader and converges to formulate the unique coping patterns of most participants with conflicts that have arisen in the employment space. The intention is to adopt a transition strategy that combines conservatism with the use of modern features and adaptation to Western work patterns. In fact, ultra-Orthodox academics are becoming the mediators between the enclave and the “foreign” population.

To sum up, it seems that, paradoxically, out of conservatism and the need for coping with those in a secular space, an innovative model of academic conservatives joining modern and advanced workplaces is emerging. The same model is worthy of development and assistance as presented in the solutions below.

## 6. Conclusions

On the basis of these findings, it seems that three main directions of action should be recommended. First, a psycho-educational program should be developed, during their academic studies, toward the integration of the ultra-orthodox academics into the employment space. The program will help strengthen the inner world of those who integrate and reduce tensions and fears of personal identity. In addition, the program will pay special attention to building effective coping strategies when joining the world of work. Furthermore, the program will accompany that group in the employment space in the early stages of entering the workplace, monitor their dilemmas and difficulties, and provide answers by way of the resources and coping strategies found to be effective in research. (Adaptive strategies include acceptance strategy, active strategy, and instrumental support.)

Creating a work support service system, in the early years of work, will help solve the professional difficulties brought up by participants during their work. This recommendation is related to the phrase “occupational disability,” conceived by one of the research participants to illustrate the difficulty of coping with one of the main barriers to ultra-Orthodox society in integration into academia and employment: lack of English proficiency. This gap is connected to a wider lack of knowledge, especially amongst ultra-Orthodox men. Therefore, it is recommended to incorporate those who intend to go to work in pre-employment preparation courses for obtaining tools and skills for optimal integration, and to assist them by providing professional knowledge as a tool for work, with emphasis on English studies and working with computers.

The third idea is aimed at developing, in cooperation with employers, employment training when the ultra-Orthodox employee is already working. Since employers in the economy have discovered the potential of the ultra-Orthodox workforce, both in terms of high output and in terms of the moral values, it is advisable to continue developing employment training programs for academic ultra-Orthodox graduates, led by employers and government encouragement. These training programs will improve the quality of work of the ultra-Orthodox workforce in reducing conflicts and tension in the workplace and will of course greatly contribute to the Israeli economy.

### Study Limitations

At the end of the present study, I can point to three methodological limitations that should be addressed in future studies. First, we propose to conduct a more comprehensive study that will also include the population of employers and will reflect the integration process from their perspective. The second refers to the examination of ultra-Orthodox society as one unit regarding their integration into the workforce. In our opinion, it would be desirable to examine this phenomenon among each ultra-Orthodox stream separately in order to better understand the way these societies struggle with the important trend discussed in this paper. The third and last recommendation is a comparison of the ultra-Orthodox community to another conservative religious group outside of Israeli society. Such a comparison is likely to bring a wider perspective to processes of change among traditional religious women who seek an education and intend to enter the workforce.

## Figures and Tables

**Table 1:** Participants in focus group for women—personal data.

Name of Participant	Community Membership	Age	Family Status	Children	Place of Residence	Profession	Place of Employment
Ronit	Lithuanian	23	Married	✓	Jerusalem	Educational Advisor	Ulpana
Sarah	Sephardic	21	Single	-	Jerusalem	Accountant	Accounting office
Gila	Outsider	34	Married	✓	Jerusalem	Doctoral Student—Biology	University
Ruti	Hassidic	29	Married	-	Jerusalem	Clinical Psychology	Psychological Services
Yaffa	Sephardic	23	Married	✓	Jerusalem	Accountancy and Information Systems	Ministry of Education
Rachel	Lithuanian	32	Married	✓	Jerusalem	Computer Programmer	Finance Ministry
Hodaya	Sephardic	28	Married	✓	Jerusalem	Computer Sciences	Government Ministry
Ora	Outsider	28	Married	✓	Jerusalem	Accountancy and Information Systems	Accounting Office
Shifra	Modern	24	Single	✓	Jerusalem	Accountancy and Business Management	Accounting Office
Shoshana	Hassidic	22	Single	✓	Jerusalem	Professional Trainer—Gymnastics	Primary School
Miriam	Hassidic	21	Married	✓	Jerusalem	Professional Trainer—Gymnastics	Center for People with Special Needs
Dvorah	Sephardic	43	Married	✓	Jerusalem	Conflict Resolution and Management	Jerusalem Municipality
Yehudit	Sephardic	21	Married	-	Jerusalem	Lifeguard and Swimming Coach	Private Framework
Bayli	Hassidic	22	Married	-	Jerusalem	Degree in gymnastics, Wingate	Private Framework
Bluma	Lithuanian	22	Married	-	Jerusalem	Professional Training—Graphics	Advertising Agency

## References

[1] Sivan E., Almond G.A., Appleby S.R. (2004). Modern Religious Fanaticism: Judaism, Christianity, Islam, Hinduism.

[2] Douglas M. (1978). Cultural Bias.

[3] Barzilai-Nahon K., Barzilai G. (2005). Cultured Technology: The Internet and Religious Fundamentalism. Inf. Soc..

[4] Stadler N. (2005). Fundamentalism. Modern Judaism: An Oxford Guide, Nicholas de Lange and Miri Freud-Kandel.

[5] Rashi T., Campbell H. (2013). The Kosher Cell Phone in ultra-Orthodox Society: A Technological Ghetto within the Global Village. Digital Religion: Understanding Religious Practice in New Media Worlds.

[6] Campbell H. (2010). When Religion Meets New Media.

[7] Cahaner L. (2018). Space, society and community: The spatial structure of the ultra-Orthodox community in Israel in an era of change. J. Law Soc. A.

[8] Lazarus R.S., Folkman S. (1984). Stress, Appraisal and Coping.

[9] Herzog H., Cohen S.A., Shein M. (2000). The Status of Women in Israel: A Fifty Years Perspective. Israel Culture, Religion and Society 1948–1998.

[10] Fuchs H., Weiss A. (2018). Education and employment of Arab youth. Taub Jerusalem: Center for Social–Policy Research in Israel.

[11] Harel-Shalev A., Kook R., Yuval F. (2018). Gender Relations in Bedouin Communities in Israel: Local Government as a Site of Ambivalent Modernity.

[12] Kalagy T., Braun-Lewensohn O. (2017). ‘Outside Versus Inside’: The Integration of Ultra-Orthodox Academics in the Israeli Workforce.

[13] Collins R. (1979). The Credential Society.

[14] Brown-Hoizman I. (2012). “I Shall Work”: Ultra-Orthodox Women Shouldering the Burden of Breadwinning: Its Justifications and Consequences. Democr. Cult..

[15] Malach G., Cahaner L. (2018). The Yearbook of Ultra-Orthodox Society in Israel 2018.

[16] Kalagy T., Braun-Lewensohn O. (2018). Agency of preservation or change: Ultra-Orthodox educated women in the field of employment. Community Work Fam..

[17] Carver C.S., Scheier M., Weintraub J.K. (1989). Assessing coping strategies: A theoretically based approach. J. Personal. Soc. Psychol..

[18] Vogt W.P. (2005). Ceiling Effect. Entry, Dictionary of Statistics and Methodology: A Nontechnical Guide for the Social Sciences, 3d ed..

[19] Atkinson R., Flint J. (2001). Accessing hidden and hard-to-reach populations: Snowball research strategies. Soc. Res. Update.

[20] Watters J., Biernacki P. (1989). Targeted sampling: Options for the study of hidden populations. Soc. Probl..

[21] Denzin N., Lincoln Y., Denzin N.K., Lincoln Y.S. (2000). The Discipline and Practice of Qualitative Research. Handbook of Qualitative Research.

[22] Bogdan R., Biklen S.K. (1998). Qualitative Research for Education: An Introduction to Theories and Methods.

[23] Corbin J., Strauss A.L. (2008). Basics of Qualitative Research.

[24] Berg L.B., Lune H. (2011). Qualitative Research Methods for the Social Sciences.

[25] Kook R., Harel-Shalev A., Yuval F. (2019). Focus Groups and the Collective Construction of Meaning: Listening to Minority Women in Israel. Women’s Stud. Int. Forum.

[26] Wasserman V., Frenkel M. (2019). The politics of (in) visibility displays: Ultra-orthodox women manoeuvring within and between visibility regimes. Hum. Relat..

